# The Physiology, Pathophysiology, and Clinical Implications of Fluid Resuscitation in Critically Ill Patients

**DOI:** 10.7759/cureus.104736

**Published:** 2026-03-05

**Authors:** Akram M Eraky, Yasser Mokhtar, Adnan Khan, Amr Farag, Sondos Elawany, Walaa Alsabbagh, Alisha Wright, Mostafa Alamrosy

**Affiliations:** 1 Department of Emergency Medicine, Freeman Health System, Joplin, USA; 2 Department of Graduate Medical Education, Kansas City University of Medicine and Biosciences, Kansas City, USA; 3 Department of Critical Care Medicine, Freeman Health System, Joplin, USA; 4 Department of Anesthesiology and Critical Care Medicine, University Hospitals Birmingham NHS Foundation Trust, Birmingham, GBR; 5 Department of Emergency Medicine, University Hospitals Birmingham NHS Foundation Trust, Birmingham, GBR; 6 Department of Internal Medicine, Northern General Hospital, Sheffield, GBR; 7 Cardiology and Angiology Unit, Department of Clinical and Experimental Internal Medicine, Medical Research Institute, Alexandria University, Alexandria, EGY

**Keywords:** colloids, crystalloids, dka, fluid resuscitation, hypervolemia, lactated ringer, normal saline, rhabdomyolysis, sepsis, shock

## Abstract

One of the most common interventions in the ED and ICU to restore physiologic homeostasis is fluid administration. It is essential for the intensivists and ED physicians to be aware of the physiological characteristics of different fluids and the potential harm associated with their use. In this article, we explore the physiology and pathophysiology of fluid administration in humans, emphasizing how fluid administration can alter acid-base balance, perfusion, osmotic and oncotic pressures, immunologic and inflammatory responses, and hemodynamics. We also discuss fluid resuscitation strategies across common clinical scenarios, such as septic shock, diabetic ketoacidosis, rhabdomyolysis, acute pancreatitis, and acute brain injury. The most recent guidelines, clinical trials, and observational studies on fluid resuscitation were discussed and critically analyzed. We also discussed fluid management in high-risk populations, such as patients with end-stage renal disease or congestive heart failure, who are particularly susceptible to hypervolemia.

## Introduction and background

Fluid resuscitation began as a rational approach to replace the fluid loss through bleeding or gastrointestinal losses. In the 17th century, the earliest proposed fluid for resuscitation was animal blood. However, adverse reactions led to its ban for human use under the decree of Châtelet in 1668. In the early 19th century, James Blundell reintroduced blood transfusion, this time using human rather than animal blood. Human blood transfusion for resuscitation then became widely used in Europe and, to a lesser extent, in North America. Nevertheless, reports of adverse effects caused blood use to decline significantly by the end of the 19th century, before the discovery of the blood antigens and antibodies. As dissatisfaction with blood transfusions grew, milk was tested as an alternative during the cholera pandemic in Toronto, Canada, for the first time, and subsequently gained popularity in North America. Ultimately, milk transfusion was discontinued due to recurrent adverse effects reported and the advent of the use of intravenous isotonic saline in fluid resuscitation [1].

**Figure 1:**
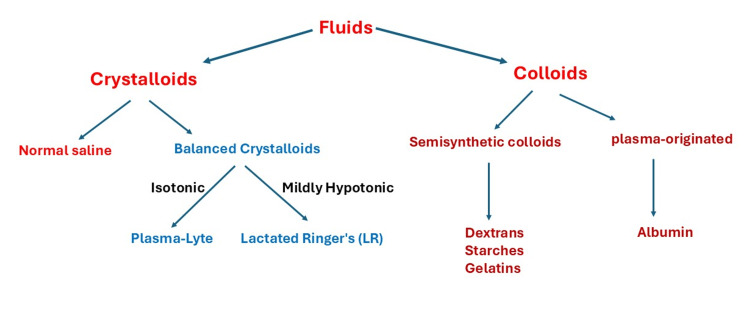
Types of resuscitation fluids Figure created by Akram M. Eraky using Microsoft PowerPoint (Microsoft Corporation, Redmond, WA, USA)

Fluid resuscitation is commonly used in critically ill patients to increase blood pressure and perfusion by increasing preload, stroke volume, and cardiac output. Resuscitation fluids are categorized into two families: crystalloids and colloids. Crystalloids are electrolytes in water that can cross the vascular barrier to the interstitial tissue. Crystalloids are considered the first-line treatment in the resuscitation of critically ill patients. Crystalloids include isotonic saline (also known as normal saline, NS) and balanced crystalloids, such as Lactated Ringer’s (LR), Hartmann’s solution, and Plasma-Lyte. On the other hand, colloids consist of large molecules that cannot penetrate capillary membranes. Colloids are classified into (1) human plasma-originated colloid, which is albumin, and (2) semisynthetic colloids, such as dextrans, starches, and gelatins (Figure 1) [2,3].

Crystalloids versus colloids

Theoretically, colloids could be more effective than crystalloids in expanding the intravascular space, as they remain mainly intravascularly; subsequently, less volume would be transferred to the extracellular space compared to crystalloids. This means a smaller volume of colloids may have the same effect as a larger volume of crystalloids. Also, complications related to extravascular fluid expansion, such as pulmonary edema, are theoretically associated with crystalloids. On the other hand, one disadvantage of using colloids is that they are more expensive and less readily available than crystalloids. Additionally, using colloids originating from human plasma might cause a transfusion reaction and anaphylactic shock [2-4]. The question here is whether, compared to crystalloids, the benefit of colloids is clinically and statistically significant. Another question is whether colloids are beneficial for a specific group of patients. These issues are discussed further in the Discussion section.

Isotonic saline versus balanced crystalloids

Historically, the cholera epidemic stimulated the scientific community to search for an appropriate solution for resuscitation. In 1832, the British physician Latta first administered intravenous water and salt solutions to treat dehydration. However, high doses of Latte’s solution were toxic, likely because the appropriate portions of electrolytes in plasma were unknown at that time. To use in the Dutch chemist Hamburger’s in vitro experiments, Hamburger created 0.9% NS in 1896. This solution was later used in animal studies and then in humans. In the 19th century, the British physician Sidney Ringer added calcium and potassium to a saline solution, creating a solution similar to extracellular fluid. Around 1932, the American pediatrician Hartmann added sodium lactate to Ringer’s solution to treat infants with diarrhea and dehydration, creating a more alkaline solution to decrease acidosis. This solution is now known as LR [5-8].

Crystalloids can be classified into (1) isotonic saline or NS, which contains an equal concentration of sodium and chloride (154 mmol/L of each), and (2) balanced crystalloids, also known as physiologic crystalloids, which contain nearly plasma-like concentrations of chloride, sodium, and potassium. In balanced crystalloids, chloride ions are replaced with a buffer that is metabolized to bicarbonate, such as lactate or acetate, or with a buffer that is excreted, such as gluconate. Of interest, bicarbonate is not used as a buffer in balanced crystalloids because the bicarbonate-containing solutions in plastic containers are not stable [2,3,9].

This difference in chemical composition between NS and balanced crystalloids results in different strong ion differences (SID). NS has a lower SID and high chloride concentration; subsequently, large volumes of NS may cause hyperchloremic metabolic acidosis. In contrast, balanced crystalloids have higher SID and may cause metabolic alkalosis. Balanced crystalloids are also relatively hypotonic compared to NS, as they contain a low sodium concentration [2,3,10-13].

## Review

Choosing the appropriate type and rate of fluid administration is crucial for guiding resuscitation in critically ill patients, as it can significantly impact patients’ overall mortality. Herein, we discuss the use of various fluids during resuscitation in different pathologies. Additionally, we will review the appropriate fluid volumes and rates in multiple pathologies, such as diabetic ketoacidosis (DKA), rhabdomyolysis, septic shock, cirrhosis, hemorrhagic shock, acute pancreatitis, traumatic brain injury (TBI), and hepatorenal syndrome (HRS). We are also discussing fluid resuscitation in special populations, such as patients with congestive heart failure (CHF) and end-stage renal disease (ESRD), as those patients may not tolerate fluid resuscitation like healthy individuals.

Colloids versus crystalloids in critically ill patients

Theoretically, the capability of colloids to expand the intravascular volume by increasing intravascular oncotic pressure is appealing, as it facilitates fluid shift from interstitial fluid to the intravascular space at the capillary level [2,3,14,15]. However, clinical trials did not demonstrate a significant advantage of colloids over crystalloids in critically ill patients, as discussed below. Additionally, colloids are more expensive and less available compared to crystalloids.

In the Saline versus Albumin Fluid Evaluation (SAFE) randomized controlled trial (RCT), there was no significant difference between the NS group and the 4% albumin group in terms of ICU stay, duration of hospital stay, days of mechanical ventilation, days of renal replacement therapy (RRT), morbidity, or mortality during the first 28 days in critically ill patients in general [16].

In the Colloids Versus Crystalloids for the Resuscitation of the Critically Ill (CRISTAL) trial, there was no significant difference between crystalloids, including NS and LR, and colloids, including albumin, gelatins, and hydroxyethyl starches, in 28-day mortality and 90-day mortality in patients with sepsis. In the overall population, including patients with sepsis, trauma, or hypovolemic shock without sepsis or trauma, there was no significant difference in mortality at 28 days, but mortality at 90 days was significantly less in the colloid group [17]. This study included only patients with hypovolemia. Patients with hypervolemia and those on hemodialysis (HD) were excluded from the study. Of interest, the administration of colloids was significantly associated with more days without mechanical ventilation within seven and 28 days compared to crystalloids. Colloids were also associated with more vasopressor-free days within seven and 28 days [17].

Notably, subgrouping patients by the type of colloid or crystalloid they received revealed a significantly lower 90-day mortality rate only in the HES group compared to the NS group. Other groups, containing patients who received albumin, did not experience lower mortality rates [17]. However, in the Crystalloid vs Hydroxyethyl Starch Trial (CHEST), a larger RCT, no significant difference in 90-day mortality was observed between HES and NS. HES was also associated with more adverse effects compared to NS [18]. In another RCT, HES was found to be associated with higher 90-day mortality and RRT compared to LR in patients with severe sepsis [19].

This discrepancy in the potential benefit of colloids between the CRISTAL trial and other trials necessitated a meta-analysis of the clinical trials [17-20]. In a Cochrane systematic review and meta-analysis, there was no significant difference between albumin or fresh frozen plasma (FFP) and crystalloids in mortality within 90 days, within 30 days, or at the end of follow-up. There was also no significant difference in the need for blood transfusion or RRT between albumin or FFP and crystalloids [21]. In the same Cochrane review, there was no significant difference in mortality within 90 days, within 30 days, or at the end of follow-up between starches and crystalloids, gelatins and crystalloids, or dextrans and crystalloids. Of interest, starches were associated with a mild increase in the need for blood transfusion and RRT [21].

In summary, there is no significant evidence that colloids have more benefits over crystalloids in the fluid resuscitation of critically ill patients in general. Given colloids’ high cost and low availability, crystalloids are recommended as the fluid of choice in the resuscitation of critically ill patients by the European Society of Intensive Care Medicine (ESICM) [22]. In the next section, we will discuss the most commonly used colloid for fluid resuscitation in the ICU: albumin.

Albumin in critically ill patients

Albumin is considered the most commonly used colloid in fluid resuscitation. It is a human plasma-derived fluid that is used for resuscitation due to its oncotic properties. Like any other blood product, albumin administration has a risk of developing transfusion-induced allergic reactions and, theoretically, prion disease. In the human body, the interstitial space contains approximately 60% of the total albumin, while the remaining albumin is found in the plasma, with a concentration of 3.5-5 g/dL [23-27].

Albumin is available in two main formulations: hyper-oncotic (20-25%) and iso-oncotic (4-5%). Sodium is added to available formulations to make them isotonic; however, hypernatremia may develop if these formulations are used for prolonged periods and at high volumes, especially in patients with preexisting hypernatremia. Additionally, according to the Gibbs-Donnan effect, the negatively charged albumin may attract the positively charged sodium ions into the extracellular space. A third formulation, known as salt-free 20% albumin, may be used in patients with preexisting hypernatremia [28,29]. Herein, we discuss the albumin effect on pharmacodynamics, coagulation, hemodynamics, and acid-base balance.

Albumin Effect on Drug Pharmacokinetics

Albumin has other physiological properties beyond its oncotic properties. It acts as a transporter of many molecules in the bloodstream, including thyroxine, amino acids, fatty acids, nitric oxide (NO), ions, and bilirubin. Albumin also interacts with many highly protein-bound medications, including phenytoin, nonsteroidal anti-inflammatory drugs, digoxin, midazolam, diazepam, and warfarin. Consequently, hypoalbuminemia may affect the effect of these medications by increasing the unbound portion of these drugs in plasma and altering their volume of distribution. As a result, hypoalbuminemia may lead to an increase in their metabolism and a decrease in their effectiveness. Of interest, these highly albumin-bound drugs are known to have a narrow therapeutic window [30-32].

Hypoalbuminemia is known to increase the unbound active portion of many medications that have highly albumin-bound activity. This can cause two paradoxical effects. First, the increased unbound active portion of the drugs may lead to an increased risk of toxicity. Second, the clearance of the unbound portion is faster, resulting in a decreased half-life [31]. Theoretically, albumin administration may have an opposite effect. It is crucial to pay attention to potential pharmacokinetic changes after albumin administration in patients who use highly albumin-bound medications. Increasing albumin levels may decrease the free, unbound portion of these drugs, thereby decreasing their metabolism and increasing their half-life, and may also result in subtherapeutic drug concentrations.

Albumin Effect on Coagulation and Its Use in Hemorrhagic Shock and Perioperatively

The effect of albumin on coagulation is unclear. Some studies show that albumin administration may reduce coagulation factor levels and affect platelet aggregation, thereby stimulating an anticoagulant effect. In contrast, other studies show that albumin may also have a procoagulant impact by lowering the levels of natural anticoagulants, such as antithrombin, protein S, and protein C; consequently, it induces microthrombus formation and ischemic injury [33-40].

Albumin concentration in plasma also affects the effectiveness of anticoagulants. Hypoalbuminemia was found to be associated with a high risk of bleeding from warfarin. This can be because hypoalbuminemia increases the unbound active warfarin in the plasma and subsequently increases the risk of developing a high international normalized ratio of prothrombin time and bleeding risk. At the same time, the increased level of unbound warfarin may increase its clearance and decrease its half-life [31]. Theoretically, albumin administration may have an opposite effect.

In the SAFE trial, 4% albumin was associated with higher mortality compared to NS in critically ill patients with trauma during the first 28 days. However, this was not statistically significant [16,41]. In a 2024 systematic review and meta-analysis of previous RCTs, no significant difference in mortality, acute kidney injury (AKI) incidence, blood loss, or duration of ICU stay was found between the albumin group and other fluids, including crystalloids, in patients undergoing cardiovascular surgery [42]. In a 2022 large clinical trial including 1386 patients, 4% albumin was found to be associated with high rates of bleeding compared to LR in patients undergoing cardiac surgery with cardiopulmonary bypass [43]. In a 2025 trial including patients undergoing high-risk cardiac surgeries, 20% albumin was associated with more blood transfusions and AKI compared to usual care [44]. None of the previous studies show a significant benefit of albumin over other fluids perioperatively or in trauma patients. In contrast, it indicates that albumin may have a potential harm in this population.

The French Society of Anesthesia and Intensive Care Medicine (SFAR) and the French Society of Emergency Medicine (SFMU) do not recommend the use of colloid solutions for fluid resuscitation over crystalloids to reduce mortality and/or the need for RRT in patients with hemorrhagic shock [45]. The ESCIM suggests the use of crystalloids over albumin in critically ill patients perioperatively and in patients with bleeding or at risk of bleeding [22]. Given the uncertain mortality of albumin administration in trauma patients and perioperatively and its equivocal effect on coagulation parameters, clinicians should avoid the routine use of albumin administration as their first choice in trauma patients and perioperatively.

Effect of Albumin Administration on Acid-Base Balance and Its Use in Acidotic Patients

**Figure 2:**
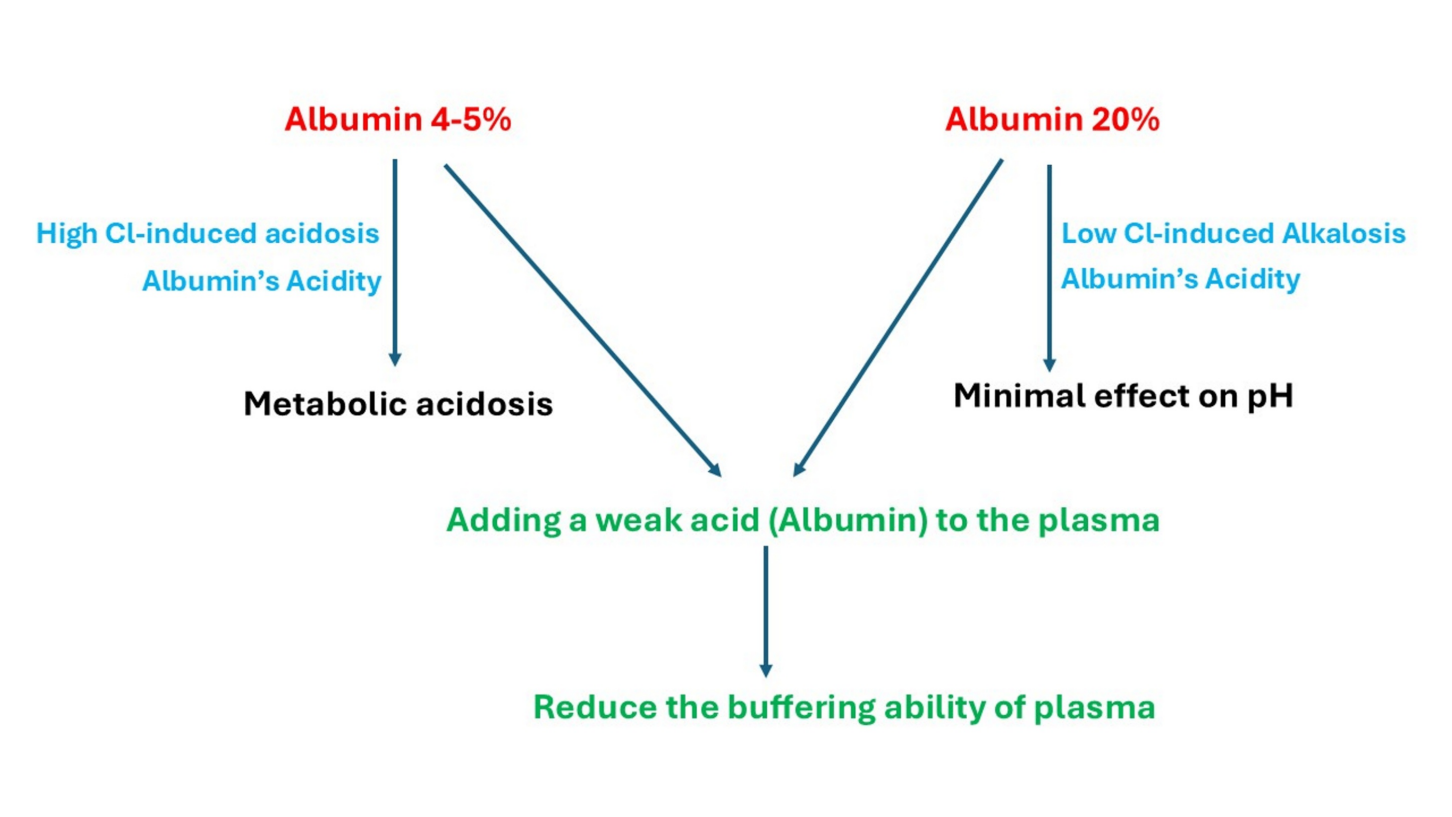
Albumin effect on acid-base balance Iso-oncotic 4-5% albumin may induce acidosis through albumin's acidity and the high chloride content of the isotonic albumin. In contrast, hyperoncotic 20% albumin has a minimal effect on the pH because of its low chloride-induced alkalosis, which offsets the effect of albumin’s acidity. Moreover, adding albumin to a solution limits its buffering ability. Figure created by Akram M. Eraky using Microsoft PowerPoint (Microsoft Corporation, Redmond, WA, USA)

Albumin is considered a weak acid with a negative charge. It was considered a buffering agent due to its negative charge and its ability to bind positively charged hydrogen ions [46,47]. However, according to Stewart’s theory, increasing the concentration of a weak nonvolatile acid, such as albumin, is expected to reduce the buffering ability of plasma. This concept was tested in an in vitro experiment by Krbec et al., who found that increasing albumin concentration in a solution makes it more acidic and less resilient against changes in pH in response to changes in pCO₂. This means adding albumin to a solution limits its buffering ability (Figure 2) [47,48].

Albumin can also contribute to metabolic acidosis because albumin solutions, particularly iso-oncotic 4-5% albumin, are rich in chloride, which can lead to hyperchloremic metabolic acidosis (Figure 2). The Fluid Expansion as Supportive Therapy (FEAST) trial found that administering 5% albumin solution in boluses increased four-week mortality in critically ill African children with impaired perfusion, compared to maintenance fluids [49]. A secondary analysis of the FEAST trial indicated that boluses of either albumin or saline were linked to hyperchloremic metabolic acidosis [50]. In a retrospective study of adult patients with sepsis, 4% albumin was found to be associated with the development of hyperchloremia and metabolic acidosis. Additionally, 4% albumin administration was associated with hypocalcemia, likely due to albumin binding to free calcium [51,52].

In contrast to iso-oncotic 4% albumin, which has a sodium concentration of 140 mmol/l and a chloride concentration of 128 mmol/l, hyperoncotic 20% albumin has a similar concentration of sodium and a chloride concentration of 65 mmol/l. The low chloride concentration in the hyperoncotic albumin may induce hypochloremia, which can subsequently lead to metabolic alkalosis. At the same time, albumin itself is a weak acid that causes metabolic acidosis. These two paradoxical actions may theoretically cancel each other out, making albumin 20% have a lesser effect on the pH (Figure 2) [53,54].

In a prospective study, the administration of 20% albumin was associated with an increase in albumin concentration, SID, and a decrease in chloride concentration. Additionally, it resulted in an unchanged pH due to the combined effects of increased SID, which led to alkalization, and increased albumin concentration, which caused acidification. Similar to 4% albumin, 20% albumin was also associated with a low free calcium concentration [53]. In another prospective study, the administration of 20% albumin boluses to patients undergoing gynecological surgery and receiving either NS or LR was associated with a significant decrease in pH in both groups. This happened despite the albumin-associated increase in both albumin concentration and SID [54]. This can be explained by the fact that the total chloride concentration is higher when albumin is co-administered with NS or LR than when albumin is administered alone. This will make the albumin’s acidic effect stronger than the SID’s alkaline effect. In general, we may conclude that the acidic effect of 20% albumin is less than the acidic effect of 4% albumin. Additionally, it is essential to note that the various commercial albumin formulations differ in electrolyte concentration.

The SWIPE RCT compared the effects of 4% albumin to the impact of 20% albumin in the same study. In the SWIPE RCT, patients receiving albumin 20% were found to have lower levels of sodium and chloride than those who received albumin 4% [15]. This confirms the conclusion from previous studies that 20% albumin has a less pronounced effect on chloride levels and, subsequently, on pH [53-54].

Clinicians should be aware that albumin administration may affect plasma pH and buffering capacity in different ways. First, albumin is a weak acid that decreases plasma pH. Second, albumin formulations contain chloride that may induce metabolic acidosis by causing hyperchloremia. Third, albumin limits plasma buffering ability. In patients with severe acidosis, albumin should be avoided, as it may impede pH improvement by reducing plasma buffering power.

Albumin Administration in Cirrhotic Patients With Hyponatremia

Cirrhosis-induced hyponatremia may occur due to various factors. Increased levels of inflammatory markers in decompensated liver failure may lead to increased production of vasodilators, which induce hypovolemia and, in turn, activate the renin-angiotensin-aldosterone system, thereby increasing antidiuretic hormone (ADH) secretion. Increased levels of ADH and aldosterone at the same time may induce hypervolemic hyponatremic status. Moreover, low albumin levels may lead to a decrease in the concentration of the negatively charged proteins, resulting in a reduction of the concentration of the positively charged sodium ions, as illustrated by the Gibbs-Donnan effect [55-58].

Albumin may correct liver cirrhosis-induced hyponatremia in many ways. First, it increases circulating volume and clears inflammatory factors. As a result, it may decrease vasodilator production and the subsequent hormonal changes. Second, the negatively charged albumin may attract the positively charged sodium ions, causing them to move into the extracellular space, according to the Gibbs-Donnan effect [55,58].

In a recent meta-analysis of two clinical studies, albumin infusion was found to be significantly associated with a lower incidence of hyponatremia and higher serum sodium levels in cirrhotic patients without hyponatremia. Regarding cirrhotic patients with hyponatremia, albumin infusion was found to be associated with a higher rate of hyponatremia resolution compared to those who did not receive albumin [55,59,60]. In another meta-analysis of three clinical studies, albumin infusion was found to be associated with a lower 30-day mortality in patients with hyponatremia [58,61].

According to the European Association for the Study of the Liver (EASL), albumin administration can be suggested in cirrhotic patients with hypervolemic hyponatremia. Also, fluid restriction to 1 L per day is recommended in cirrhotic patients with hypervolemic hyponatremia. In contrast, administration of NS is recommended in cirrhotic patients with hypovolemic hyponatremia [62].

According to the American Association for the Study of Liver Diseases (AASLD) 2021 guidelines, mild hyponatremia (126-135 mEq/L) in cirrhotic patients without symptoms only requires monitoring and water restriction. In moderate hyponatremia (120-125 mEq/L), water restriction to 1 L/day and cessation of diuretics are recommended. In severe hyponatremia (less than 120 mEq/L), albumin infusion with water restriction is recommended [63].

Albumin Administration in Critically Ill Patients With CHF and Hypoalbuminemia

Low albumin levels are common in CHF patients due to hepatic congestion, intestinal congestion-induced malabsorption, malnutrition, suppressed albumin synthesis, and hypervolemia-induced hemodilution. Low albumin levels were found to be associated with increased mortality in CHF patients due to the loss of albumin protection against oxidative stress and the low intravascular oncotic pressure-induced interstitial congestion [64-67]. Theoretically, albumin administration may reduce these undesired effects of low albumin levels. However, recent studies show that albumin administration in critically ill patients with low albumin and CHF may increase mortality [68,69].

In a retrospective study by Li et al., albumin administration in critically ill patients with CHF and hypoalbuminemia (serum albumin < 3.4 g/dL) was associated with increased mortality, ICU length of stay (LOS), and hospital LOS [69]. In another study by Yao et al., albumin administration in critically ill patients with CHF and hypoalbuminemia (serum albumin < 3 g/dL) was associated with increased in-hospital mortality, ICU LOS, and hospital LOS. This association was not found in patients with serum albumin levels above 2.9 g/dL [68]. This could be due to the unexpected effect of albumin administration in septic and critically ill patients. Serum albumin levels in septic patients decreased more rapidly than those of healthy controls after albumin administration due to increased capillary leakage [70]. The increased capillary leakage in septic patients shifts the administered albumin from the capillaries to the interstitial space, resulting in potential worsening symptoms in CHF patients. Physicians should be cautious about albumin administration in critically ill patients with CHF and hypoalbuminemia.

Albumin Administration in Cirrhotic Patients With Hepatic Encephalopathy (HE)

HE is a life-threatening complication in cirrhotic patients. It develops as a consequence of the accumulation of high levels of nitrogen species and reactive oxygen particles [71,72]. Theoretically, albumin infusion could have an anti-inflammatory effect by reducing oxidative stress and the production of inflammatory cytokines. In an RCT, albumin was found to decrease the levels of inflammatory cytokines more effectively than the usual treatment alone [71].

In an RCT by Sharma et al., patients with overt HE treated with albumin and lactulose were found to have lower mortality rates, shorter hospital stays, and a greater decrease in serum cytokine and ammonia levels compared to those who received lactulose alone [71]. In a previous RCT, patients with overt HE treated with albumin, in addition to the usual treatment, had a better survival rate at day 90 than those who received saline. However, the HE resolution on day 4 did not differ significantly between the two groups [73]. In three meta-analysis studies, albumin infusion was found to be associated with reduced mortality in cirrhotic inpatients with HE [72,74,75].

In the outpatient settings, albumin infusions were also found to be beneficial in cirrhotic patients with prior HE or current mild HE. In the Hepatic Encephalopathy Albumin (HEAL) trial, weekly albumin infusions were found to be effective in improving cognitive function in cirrhotic outpatients with prior HE or current mild HE [76]. Physicians should consider albumin administration in cirrhotic inpatients with overt HE to improve mortality.

Albumin Administration in Cirrhotic Patients Undergoing Large-Volume Paracentesis (LVP)

Paracentesis-induced circulatory dysfunction (PICD) is a post-paracentesis hyperdynamic condition that results from the removal of a large volume of ascitic fluid, leading to a drop in peripheral vascular resistance and mean arterial pressure (MAP), which in turn activates the renin-angiotensin system (RAS). As a result, RSA and sympathetic system activation may lead to renal dysfunction and increased mortality. PICD is also associated with fast ascites recurrence and hyponatremia. It happens in up to 80% of patients undergoing LVP without receiving plasma expanders. It may also develop progressively over up to 6 days [77,78]. Theoretically, albumin administration may serve as an effective long-term plasma expander, eventually preventing MAP deterioration, RAS activation, and PICD. Many clinical trials and meta-analyses showed that albumin administration after LVP decreases the incidence of PICD [77,79-84]. Many guidelines support the use of albumin in cirrhotic patients after LVP to prevent PICD, as discussed below.

The British 2020 guidelines recommend administering 8 g of 20-25% albumin for every liter of removed fluid after paracentesis if the removed ascitic fluid volume exceeds 5 L to prevent PICD, as a strong recommendation with high-level evidence. After a paracentesis of less than 5 L of ascitic fluid, they also recommend considering albumin administration in patients with AKI or acute-on-chronic liver failure (ACLF) as a weak recommendation with a low level of evidence [85].

The AASLD 2021 guidelines recommend albumin infusion in LVP of more than 5 L with a dosage of 6-8 g of albumin for every liter of the removed ascitic fluid [63]. Additionally, the SFAR and the French Association for the Study of the Liver (AFEF) recommend administering 6-8 g of 20-25% albumin per liter of the removed ascitic fluid if the removed ascitic fluid volume exceeds 4-5 L to prevent PICD [86]. The 2024 International Collaboration for Transfusion Medicine Guidelines (ICTMG) recommends the use of albumin in cirrhotic patients undergoing LVP (>5 L) as a conditional recommendation with very low certainty of evidence of effect [87]. All of the previous guidelines recommend the use of albumin in cirrhotic patients after LVP to prevent PICD.

Albumin Administration in Cirrhotic Patients With Spontaneous Bacterial Peritonitis (SBP)

In cirrhotic patients with SBP, increased production of cytokines and NO leads to splanchnic vasodilatation, which subsequently stimulates the RAS and worsens the hyperkinetic state. This increases the risk of developing renal function impairment [88-92]. Serum and ascitic fluid levels of NO were also found to be associated with the development of kidney injury in cirrhotic patients with SBP [93]. Albumin is thought to be beneficial in cirrhotic patients with SBP by two mechanisms. First, it can reduce plasma levels of inflammatory cytokines, such as TNF-α and IL-6, as well as endotoxin levels in ascitic fluid. Additionally, it can reduce the NO levels in both ascitic fluid and plasma [88-90]. Furthermore, albumin may decrease the plasma renin activity, creatinine levels, and heart rate. Albumin was also found to increase the peripheral vascular resistance and MAP [94].

Due to its immunological and hemodynamic benefits, as well as its potential to prevent renal impairment and improve survival, many clinical trials have discussed the use of albumin in cirrhotic patients with SBP. Many clinical trials and meta-analysis studies showed that the use of albumin in cirrhotic patients with SBP improved mortality and decreased the incidence of renal impairment [94-96]. Many guidelines support the use of albumin in cirrhotic patients with SBP.

The British 2020 guidelines recommend albumin administration in cirrhotic patients with SBP and an elevated serum creatinine or trending-up creatinine. Administration of 1.5 g of albumin/kg within six hours of diagnosis and 1 g of albumin/kg on day 3 is recommended as a weak recommendation with a low level of evidence [85]. The AASLD 2021 guidelines recommend administering 1.5 g of albumin/kg on day 1 and 1 g/kg on day 3 in cirrhotic patients with SBP [63]. Additionally, the SFAR and the AFEF recommend albumin administration in patients with SBP [86]. According to the 2024 ICTMG, albumin administration is recommended in cirrhotic patients with SBP to decrease mortality as a conditional recommendation with low certainty of evidence of effect [87]. All of the previous guidelines recommend the use of albumin in cirrhotic patients with SBP. Table 1 summarizes the different doses of albumin in different clinical scenarios according to the AASLD.

**Table 1:** Albumin doses in different clinical scenarios by the AASLD 2021 guidelines AASLD, American Association for the Study of Liver Diseases; HRS, hepatorenal syndrome; LVP, large-volume paracentesis; SBP, spontaneous bacterial peritonitis Source: Biggins et al. (2021) [63]

Indication	Dosage
LVP	6-8 g of albumin per liter of ascitic fluid removed
SBP	1.5 g/kg on day 1 and 1 g/kg on day 3
HRS	1 g/kg on day 1, followed by 40-50 g/day for the remainder of therapy

Albumin Administration in Cirrhotic Patients With Extraperitoneal Infection

In contrast to the use of albumin administration in cirrhotic patients with SBP, albumin administration in cirrhotic patients with extraperitoneal infection is more controversial and has less evidence. Theoretically, albumin administration in patients with extraperitoneal infections may improve mortality due to its antioxidant effects and its role in correcting hypoalbuminemia, which is associated with increased mortality [97].

In an RCT, there was no significant difference in in-hospital mortality between cirrhotic patients with extraperitoneal infection who received albumin 20% with antibiotics and those who received antibiotics alone. In this study, patients with septic shock, severe chronic pulmonary disease, and chronic heart failure were excluded. Notably, administration of 20% albumin was associated with a higher incidence of ACLF resolution and a lower incidence of nosocomial infections [98]. This means that patients who received albumin 20% had better outcomes.

In another clinical trial, the administration of 20% albumin delayed the onset of renal failure in cirrhotic patients with extraperitoneal infections. However, it did not change the incidence of renal failure at three months [99]. In a third RCT by Guevara et al., 20% albumin was associated with improved renal function for up to 14 days compared to the control group. Albumin was also associated with lowering the levels of both renin and aldosterone at seven days, compared to the control group [100]. Interestingly, albumin administration was found to be an independent predictor of survival in cirrhotic patients with extraperitoneal infections [100]. In both RCTs, patients with septic shock were excluded from the studies.

**Figure 3:**
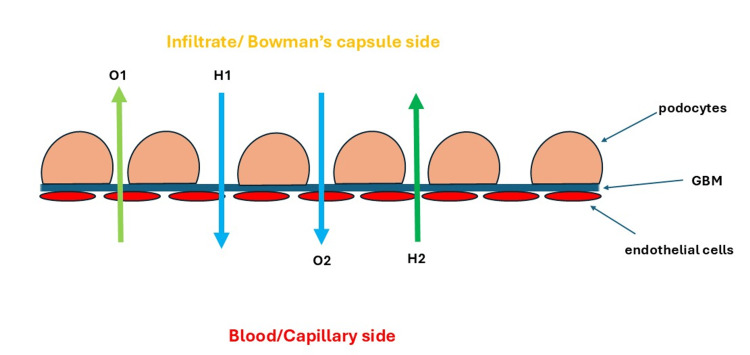
Effect of albumin administration on the GFR The RBB consists of three layers: the capillary endothelial cells, the GBM, and the podocytes. The fluid movement through the RBB is controlled by four forces: the oncotic pressure in Bowman’s capsule (O1), the hydrostatic pressure in Bowman’s capsule (H1), the intracapillary hydrostatic pressure (H2), and the oncotic pressure in the intrarenal capillaries (O2). The net filtration = (H2+O1) - (H1+O2). Albumin administration might increase O2, thereby theoretically negatively affecting net filtration and subsequently decreasing the GFR. However, albumin also increases the intracapillary hydrostatic pressure (H2), which increases the net filtration rate. GFR improvement through albumin administration might occur because its H2 is more potent than its O2. GBM, glomerular basement membrane; GFR, glomerular filtration rate; RBB, renal blood barrier Figure created by Akram M. Eraky using Microsoft PowerPoint (Microsoft Corporation, Redmond, WA, USA)

These two previous trials show that albumin administration has a beneficial effect on renal function. However, the trial by Thévenot et al. shows that this beneficial effect on renal function is only observed during the early stages, as the incidence of renal impairment is similar at three months. Also, the trial by Guevara et al. checked renal impairment only up to 14 days [99,100]. Theoretically, albumin may negatively affect the glomerular filtration rate (GFR) by increasing the blood’s oncotic pressure. However, many studies have shown that albumin may improve the GFR, likely because it also increases the blood hydrostatic pressure, which has a more substantial effect on the GFR and offsets the adverse impact of albumin’s oncotic pressure (Figure 3).

Two systematic reviews and meta-analyses of the previous three trials show that there is no significant difference between the albumin group and the control group in terms of renal impairment and mortality in cirrhotic patients with extraperitoneal infections [101,102]. However, albumin administration was found to be associated with a higher incidence of pulmonary edema [102]. This suggests that albumin may not affect renal function, but it increases the risk of developing pulmonary edema.

The ICTMG does not suggest the use of albumin to reduce kidney failure or mortality in cirrhotic patients with extraperitoneal infections, with a very low certainty of evidence of effect [87].

Albumin Administration in Cirrhotic Patients With Septic Shock

Theoretically, administering albumin in septic shock resuscitation is appealing because its ability to increase oncotic pressure and subsequently increase intravascular volume requires less volume compared to crystalloids. However, it has many other undesired effects in septic patients, such as its acidotic effect, coagulopathy, and risk of developing pulmonary edema, as we discussed above.

In a retrospective study, albumin administration in septic patients with hypoalbuminemia was found to be associated with a higher 30-day mortality rate, pulmonary edema, and heart failure, compared to those who did not receive albumin [103]. It is not clear which albumin concentration was used in this population. Additionally, the level of volume depletion and hypotension in those patients is unclear. The indication for administering albumin was only to correct hemodynamics or to maintain the albumin level above a certain threshold.

In the FRISC trial, albumin 5% was found to achieve greater hypotension reversal in cirrhotic patients with sepsis-induced hypotension at one and three hours, compared to NS. Additionally, albumin 5% was found to be associated with a higher reduction in heart rate and lactate levels, as well as a higher survival rate at one week [104]. In the ALPS trial, albumin 20% was found to be superior to Plasma-Lyte in achieving MAP above 65 at three hours and decreasing lactate levels in cirrhotic patients with sepsis-induced hypotension. However, there is no significant difference in 28-day mortality or the need for dialysis between albumin 20% and Plasma-Lyte. Interestingly, albumin 20% was associated with a higher incidence of pulmonary edema compared to Plasma-Lyte [14].

Due to its rapid hemodynamic effects and lower volume requirement than crystalloids, albumin may be considered an adjunctive fluid in cirrhotic patients with septic shock. However, albumin should be used with caution, as it may cause pulmonary edema and affect acid-base balance and coagulation. Interestingly, the ESCIM suggests the use of albumin over crystalloids in critically ill patients with liver cirrhosis as a conditional recommendation with a very low certainty of evidence [22].

Albumin Administration in Critically Ill Patients With Septic Shock

In the SAFE trials, no difference was found between NS and 4% albumin regarding mortality in septic patients [16]. Similarly, in the CRISTAL trial, there was no difference in mortality between septic patients who received albumin and those who received NS [17]. Other trials showed the same result [105,106]. Also, numerous systematic reviews and meta-analyses indicate that there is no significant difference in mortality [41,107-111]. Some meta-analyses suggest that albumin could be associated with decreased mortality in septic patients [109,110,112]. Only one meta-analysis study found a significant difference in mortality in favor of albumin over crystalloids [112]. Also, after adjusting the SAFE study for baseline covariates, albumin was found to be significantly associated with a lower odds ratio for mortality [113].

This suggests that there is no strong evidence to support the notion that albumin has a mortality benefit. Given the high albumin expense, its scarcity, and the weak evidence of its mortality benefit, crystalloids should remain the first line of fluid resuscitation. 

In the RCT by Caironi et al., there was no difference in mortality between 20% albumin and crystalloids versus crystalloids alone. Notably, the time to vasopressor suspension was shorter in the albumin group. Also, the MAP was higher in those who received albumin at six hours [106]. Other studies have shown that albumin administration is associated with a more substantial effect on hemodynamics and the use of smaller volumes compared to crystalloids [14,104]. This strong and rapid hemodynamic effect stems from the fact that albumin increases intravascular volume more effectively than crystalloids by increasing intravascular oncotic pressure, which prevents fluid from moving into extravascular and interstitial spaces. Also, it may induce fluid movement from the interstitial space into the intravascular space.

This positive effect on hemodynamics could explain the potential benefit of albumin administration on mortality, as suggested in some studies [112,113]. Also, adding albumin to crystalloids rather than using crystalloids alone should be considered in patients who require large-volume fluid resuscitation.

According to the SFAR, SFMU, ESCIM, and 2021 sepsis guidelines, crystalloids are preferred over albumin in septic patients. However, according to the 2021 sepsis guidelines, albumin is recommended for use in patients with sepsis and septic shock over crystalloids alone when large volumes of crystalloids are being administered, with a weak recommendation and moderate quality of evidence [22,45,114].

Albumin 4% Versus Albumin 20% in Critically Ill Patients Requiring High Volume of Fluids

In the SWIPE RCT, patients receiving 20% albumin required less resuscitation fluid at 48 hours than those receiving 4% albumin. Additionally, patients who received 20% albumin received less fluid volume after initial resuscitation than those who received 4% albumin. Notably, patients who received 20% albumin had lower ICU mortality than those who received 4% albumin [15]. In a retrospective study, patients who received a median volume of 500 mL of 4% albumin exhibited hemodynamic effects similar to those who received a median volume of 100 mL of 20% albumin [115]. These two studies demonstrate that 20% albumin has a greater effect on volume expansion than 4% albumin.

Albumin-Induced Pulmonary Edema

Hypoalbuminemia is associated with the risk of developing respiratory dysfunction and acute respiratory distress syndrome [116]. In addition, many animal studies show that it is associated with slow lung edema clearance [117-119]. Furthermore, hypoalbuminemia decreases the colloid osmotic pressure (COP) that subsequently increases fluid efflux from capillaries to the interstitial space and then to the alveoli. Theoretically, colloids may be administered to prevent complications of hypervolemia, such as pulmonary edema. However, colloid administration was found to induce pulmonary edema because it rapidly increases intravascular volume, leading to a subsequent rise in hydrostatic pressure that offsets the oncotic pressure’s effect, stimulating fluid efflux from capillaries into the interstitial space and then into the alveoli, as discussed below (Figure 3).

Several studies have shown that albumin administration is associated with the development of pulmonary edema [14,102,103,120,121]. This can occur due to the rapid increase in intravascular volume and the subsequent rise in intravascular hydrostatic pressure, resulting from an albumin-induced increase in intravascular oncotic pressure that pulls fluid from the interstitial spaces. Consequently, a rapid increase in preload and the hydrostatic pressure in the pulmonary capillaries leads to pulmonary edema.

This explanation is supported by studies showing that co-administration of albumin and diuretics improves oxygenation and reduces lung injury. Diuretics may counteract the strong effect of the albumin-induced rapid increase in intravascular volume, without also affecting the oncotic pressure of the administered albumin [122-124]. This suggests that the rapid rise in hydrostatic pressure is the cause of pulmonary edema, and when it subsides with diuretics, pulmonary edema does not develop. This also indicates that the alveoli are more affected by the albumin-induced increase in hydrostatic pressure than by albumin’s oncotic pressure.

**Figure 4:**
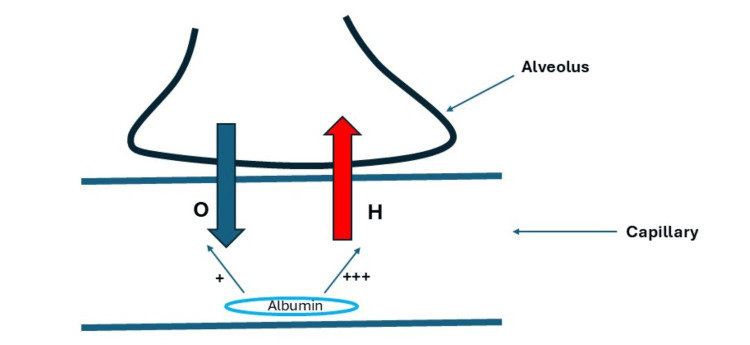
Effect of albumin administration on the development of pulmonary edema Albumin administration increases the oncotic pressure in the capillaries (O), which may theoretically decrease pulmonary edema. However, albumin also increases the intracapillary hydrostatic pressure (H), which is more potent than the increase in the intracapillary oncotic pressure. Figure created by Akram M. Eraky using Microsoft PowerPoint (Microsoft Corporation, Redmond, WA, USA)

In an RCT, the pulmonary leak index increased during fluid resuscitation, regardless of the type of fluid administered and underlying disease. The extravascular lung water (EVLW) was not affected by the type of fluid used during resuscitation in both septic and nonseptic patients. EVLW was inversely associated with the difference between COP and the central venous pressure (CVP). During fluid administration, the gradient between COP and CVP decreased and was not affected by the type of fluid [125]. This suggests that pulmonary edema is primarily influenced by hydrostatic pressure, rather than oncotic pressure or the type of fluid. Colloids increase both COP and CVP more than crystalloids. The rapid, substantial increase in CVP by colloids seems to counteract the COP effect (Figure 4).

The consensus published by the American Thoracic Society on the use of colloids in critically ill patients stated that hydrostatic pressure is more critical than osmotic pressure in the development of pulmonary edema. The consensus also stated that colloids may reduce tissue edema but could exacerbate pulmonary edema during shock resuscitation [126]. In summary, there might be a potential harm of albumin administration in critically ill patients through causing pulmonary edema. Additionally, there is a lack of RCTs demonstrating potential benefits. The ESICM guidelines suggest using crystalloids over albumin in critically ill patients with acute respiratory failure [22].

LR versus NS in fluid resuscitation

Herein, we discuss the characteristics of the most commonly used crystalloids in the ICU, including LR and NS, as well as the potential changes in human physiology after administration of different crystalloids. While NS has equal concentrations of sodium and chloride (154 mmol/L), LR has plasma-like concentrations of various ions, including potassium (4.0 mmol/L), chloride (109 mmol/L), sodium (130 mmol/L), and calcium (1.5 mmol/L). It also contains 28 mmol/L of lactate, which acts as a buffer [127,128].

Isotonic Saline-Induced Hyperchloremia and Metabolic Acidosis

Hyperchloremia, which is associated with the administration of high-chloride fluids, is thought to have many adverse effects, such as metabolic acidosis, AKI, and mortality [3,10,129]. In a retrospective study, a high chloride load, determined by the chloride ion concentration of the administered fluids and the volume of fluids, was associated with an increased risk of death over one year in patients undergoing high-volume resuscitation [130]. However, in this study, no association was found between the chloride load and the development of hyperchloremic metabolic acidosis [130]. This lack of association between high chloride fluids and the development of metabolic acidosis could be attributed to the population’s diversity, which includes variable renal ability to clear excess chloride ions in patients with different pathologies.

Another observational study showed that patients who developed postoperative AKI had lower SID and higher chloride levels compared to patients who did not develop AKI postoperatively. Also, there was a significant association between intraoperative chloride load and postoperative AKI [129]. This suggests an association between hyperchloremia and developing AKI. Lowering the chloride load may decrease the incidence of hyperchloremia and attenuate the development of AKI.

Other observational studies have suggested an association between high chloride fluids and AKI incidence, hyperchloremia, metabolic acidosis, and mechanical ventilation time [10,131-135]. However, in other studies, AKI and metabolic acidosis were not found to be significantly associated with hyperchloremia [130,136,137]. In summary, many studies suggest potential harm from high-chloride fluids, while no studies report a definitive benefit of high-chloride fluids compared to low-chloride fluids. Therefore, it might be reasonable to avoid high chloride fluids, such as NS, especially in patients presenting with preexisting hyperchloremia [11].

In a 2025 RCT, patients who received NS had higher chloride levels and lower pH than those who received LR or Crystasol. Of interest, chloride urinary excretion was not significantly different between NS and LR. However, at the end of the trial, urinary chloride concentration was higher, and diuresis was lower in the NS group compared to the LR group [138]. These findings are consistent with Stewart’s theory. It also highlights the kidneys' limitations in counteracting the increased plasma chloride levels. This suggests that clinicians should avoid high-chloride solutions in patients with acidosis or preexisting hyperchloremia.

Balanced Crystalloids-Induced Hyperkalemia

There is concern that potassium-containing balanced crystalloids, such as LR and Plasma-Lyte, may cause hyperkalemia, especially in patients receiving HD, because these solutions contain potassium concentrations of 4 and 5 mmol/L, respectively.

In a prospective observational study, severe hyperkalemia was associated with critically ill patients who received NS compared to those who received LR [139]. In two clinical trials, LR was found to be associated with lower potassium levels than NS perioperatively in patients undergoing kidney transplantation [140,141]. In a Cochrane systematic review and meta-analysis, no significant difference was found between NS and LR in the development of hyperkalemia perioperatively in patients undergoing kidney transplantation [142]. These studies suggest that LR administration is not associated with the development of hyperkalemia. A potential explanation for the lack of a significant association between LR administration and hyperkalemia is that using potassium-containing fluids with potassium concentrations lower than the plasma potassium concentration may not be sufficient to raise potassium levels [45].

LR-Induced Serum Hypoosmolality

NS has 154 mmol/L of sodium and chloride, and its calculated osmolality is 308 mOsm/kg. However, the measured osmolality of NS is 287 mOsm/kg due to factors such as ions’ interactions [143]. The measured osmolality of NS is similar to that of human serum (275-295 mOsm/kg). In contrast, the osmolality of LR is 273 mOsm/L, which is slightly lower than the osmolality of human serum. As a consequence, rapid administration of large volumes of LR at high rates can rapidly decrease serum osmolality in patients with high osmolality and may cause cerebral edema [144]. In animal studies, high-volume LR infusion at high rates was associated with a rapid decrease in osmolality and subsequent development of cerebral edema [145-148]. In a study involving healthy volunteers, administering large volumes of LR resulted in a mild, transient decrease in serum osmolality. In contrast, large volumes of NS did not change serum osmolality [144].

LR-Induced Increased Lactate Levels

Lactate production increases at the cellular level in response to hypoxia. It plays a protective role by acting as a buffer, binding to protons and forming lactic acid. Additionally, it can be metabolized into bicarbonate. The long-held misconception that lactate is a waste product that causes cellular acidosis lacks evidence. Lactate production increases as a response to exercise, hypoxia, or septic shock as a cellular defense mechanism. Lactate is considered a consequence of acidosis and not the cause of acidosis. Subsequently, it is used as a biomarker that correlates with the severity of acidosis [149-151]. This is similar to B-type natriuretic peptide, which correlates with hypervolemia and is used as a biomarker for hypervolemia; however, it is not the cause of hypervolemia and has a protective mechanism against it [152].

LR may cause mild alkalosis as its lactate binds to protons and can be metabolized to bicarbonate. In a recent clinical trial, LR was found to cause a mild increase in serum lactate levels. However, there was no significant difference between NS and LR in increasing the lactate levels [153]. Similar results were shown in a previous clinical trial [154]. In another retrospective study, there was no difference between LR and NS in lactate clearance [155]. The fear of giving LR to patients with elevated lactate is unreasonable.

Theoretically, in patients with liver cirrhosis, hepatic lactate metabolism is impaired, and high lactate levels are expected when large volumes of LR are administered. In a secondary analysis of patients with cirrhosis from the Isotonic Solutions and Major Adverse Renal Events Trial (SMART), no significant difference in lactate levels was observed between patients with cirrhosis who received LR and those who received NS [156]. This indicates that, in patients with liver cirrhosis, administering high volumes of LR does not significantly affect serum lactate levels compared with NS.

Fluid resuscitation in different clinical scenarios

In this section, we will review fluid resuscitation in common clinical scenarios, such as septic shock, acute pancreatitis, rhabdomyolysis, DKA, TBI, and HRS.

Fluid Resuscitation in Patients With Sepsis and Septic Shock

Sepsis is a life-threatening dysregulated reaction to an infection that results in end-organ damage due to tissue hypoperfusion [157]. Immediate fluid resuscitation is crucial to reverse sepsis-induced tissue hypoperfusion. There were no guidelines or standards for sepsis management until 2001, when Rivers et al. proposed the use of Early Goal-Directed Therapy (EGDT) in septic patients. The EGDT idea is an attempt to mimic the management approach for stroke and myocardial infarction (MI) in septic patients by initiating immediate treatments, including early fluid aggressive resuscitation and early antibiotics, as a method to restore perfusion to the different organs to prevent ischemic injury. The concept of time equals tissue viability in MI and stroke was introduced for the first time in sepsis management [158].

The trial by Rivers et al. showed that EDGT is superior to usual care in septic patients. The EGDT established the basis of the Surviving Sepsis Campaign sepsis guidelines and formed the fundamentals of the quality assessment by the National Quality Forum, which the Centers for Medicare & Medicaid Services (CMS) uses to ensure quality of care in septic patients [158,159]. However, three trials, including the Australasian Resuscitation in Sepsis Evaluation (ARISE), Protocolized Care for Early Septic Shock (PROCESS), and Protocolized Management in Sepsis (PROMISE) trials, challenged the EDGT approach and did not show that EDGT is superior to standard care [160-162].

The results of these trials could be attributed to the enhanced standard care provided by more experienced physicians following the introduction of Rivers et al.’s EDGT, which encouraged early antibiotic initiation and prompt fluid resuscitation, aligning usual care more closely with established guidelines and protocols. This similarity between the guidelines and the new usual care after the spread of EDGT in routine practice makes these trials misleading [163-166]. Also, these trials included patients who were less sick, which questions their external validity [159,166]. Observational studies should be considered to validate external validity, a limitation of clinical trials in general. Many observational studies and meta-analyses support the use of EDGT [167,168].

Sepsis-induced hypoperfusion results from macrocirculatory changes, such as decreased MAP, preload, and CVP, and microcirculatory changes, such as endothelial damage and increased permeability of small capillaries in response to the cytokines and inflammatory reactions. It is easy to assess macrocirculatory changes controlling macroperfusion by using blood pressure monitoring and an echocardiogram. However, it is more challenging to evaluate the microcirculation changes that control microperfusion [169,170].

Some biomarkers, such as lactate levels and urine output, can reflect tissue microperfusion. Despite their objective role in assessing microperfusion compared to the relatively subjective skin changes, these biomarkers may take time to trend and monitor. The fastest marker to monitor the changes and severity of microperfusion, especially during initial resuscitation, is skin examination, including capillary refill time; presence and extent of the skin color changes, such as mottling; and skin temperature [169-172].

In healthy individuals, changes in macroperfusion are proportionate to changes in microperfusion. In contrast, in septic patients, improving macrocirculatory changes, such as the MAP, does not guarantee adequate improvement in microperfusion due to sepsis-induced microcirculatory changes. As a consequence, macrocirculatory parameters cannot serve as independent indicators of microperfusion resolution or reversal of organ failure. In alignment with that, many studies have found that optimizing blood pressure should not be an independent indicator of microperfusion resolution, and in sepsis, there is a dissociation between macrocirculatory changes and microperfusion, because microperfusion might persist despite optimizing macrocirculatory parameters in sepsis due to the microcirculatory changes [173-177]. Notably, microperfusion parameters and microcirculatory changes were found to be significantly associated with mortality in septic patients, as opposed to macrocirculatory changes [178].

Fluid administration in septic patients improves macroperfusion through increasing CVP, SV, MAP, and COP. Its effect on microperfusion varies due to sepsis-induced changes in microcirculation, including increased permeability and endothelial damage. Theoretically, fluid resuscitation also improves microperfusion by increasing blood flow and capillary hydrostatic pressure, especially in fluid-responsive patients. This effect of fluid administration is evident in patients with healthy microcirculation, such as patients with hypovolemic shock. In contrast, in septic patients, this effect may vary significantly depending on the extent of microcirculatory and capillary damage [179,180]. In early sepsis, there are fewer microcirculatory and microperfusion changes; subsequently, fluid administration has a stronger effect on improving microperfusion. In contrast, in late sepsis, microvascular perfusion did not improve after fluid administration due to more substantial microvascular changes. This effect was independent of the macrovascular changes, such as MAP and cardiac index [181].

Due to the stronger association between microperfusion and end-organ damage, as well as mortality, compared to macrocirculatory changes, the 2021 sepsis guidelines recommend using microperfusion parameters, such as capillary refill time and lactate levels, to guide resuscitation, in addition to other measures of perfusion [114].

Fluid type during resuscitation in patients with sepsis and septic shock: According to the SFAR, SFMU, ESCIM, and 2021 sepsis guidelines, crystalloids are preferred as a first-line fluid in the resuscitation of septic patients because albumin has a higher risk of developing pulmonary edema and worsening acidosis and also has a higher cost, as discussed earlier [14,51,52,102,103,114]. Due to albumin's rapid and substantial effect on improving hemodynamics with smaller volumes compared to crystalloids, which is attributed to albumin’s strong oncotic pressure, adding albumin to crystalloids is suggested for use in septic patients who require large volumes of fluids, according to the 2021 sepsis guidelines, as we discussed earlier [14,104,106,114].

Regarding crystalloids, theoretically, LR could be considered superior to NS in septic patients due to the potential adverse effects induced by NS, such as hyperchloremic metabolic acidosis and AKI, and the possible positive effects of LR, such as its higher pH and similarity to plasma in electrolyte concentrations [138,182-184]. Several clinical studies have demonstrated that LR is superior to NS in terms of mortality in septic patients [41,185-190].

In the Isotonic Solution Administration Logistical Testing (SALT) trial, balanced crystalloids were associated with lower mortality compared to NS in septic patients [188]. A secondary analysis of the SMART (Isotonic Solutions and Major Adverse Renal Events) trial showed that LR is associated with lower 30-day mortality and major kidney events compared to NS. LR was also associated with higher RRT-free days and the number of vasopressor-free days [185]. A secondary analysis of the Crystalloid Liberal or Vasopressors Early Resuscitation in Sepsis (CLOVERS) trial shows that LR for initial resuscitation for hypotension in septic patients is associated with a lower mortality rate compared to NS [189]. Additionally, a systematic review and sequential network meta-analyses show that balanced crystalloids are associated with a lower mortality rate than NS in septic patients [41]. Another systematic review and meta-analysis shows that NS is associated with higher mortality and AKI compared to LR [191].

In contrast, some other studies did not show a difference in mortality and kidney function between balanced crystalloids and NS [192-195]. In the Plasma-Lyte 148 versus Saline (PLUS) trial, there was no difference in mortality between balanced crystalloids and NS in critically ill patients and also in the subgroup of septic patients [193]. In the Balanced Solutions in Intensive Care Study (BaSICS) trial, there was no difference in 90-day mortality between balanced crystalloids and NS in critically ill patients, including the subgroup of septic patients [194]. This can be attributed to heterogeneity in fluid administration before enrollment in the study. In a secondary analysis of the BaSICS trial, in unplanned admissions due to sepsis, balanced crystalloids were superior to NS in terms of mortality odds ratio and probability of benefit [196].

A recent meta-analysis of RCTs demonstrated no significant difference in mortality or AKI between balanced crystalloids and NS. However, LR was associated with lower mortality compared to NS in subgroup analyses of observational studies, as well as in the pooled meta-analysis that included both RCTs and observational studies [197].

In general, current studies suggest that balanced crystalloids have potential benefits for mortality and kidney function compared to NS, consistent with the theoretical advantages of balanced crystalloids and the potential harms of NS. At the same time, some studies and clinical trials do not suggest the existence of this benefit of balanced crystalloids over NS. This indicates that the level of evidence supporting balanced crystalloids over NS is weak. However, given the small cost difference between balanced crystalloids and NS and the potential benefits of balanced crystalloids in septic patients, it is recommended to use balanced crystalloids, such as LR, rather than NS. According to the SFAR, SFMU, ESCIM, and 2021 sepsis guidelines, balanced crystalloids, such as LR, are preferred over NS for fluid resuscitation in septic patients [22,45,114].

Fluid volumes during initial resuscitation in patients with sepsis and septic shock: During initial resuscitation, the 2021 sepsis guidelines recommend administering 30 mL/kg, based on ideal body weight (IBW), of fluids to septic patients with shock or sepsis-induced hypoperfusion within the first three hours. It is noteworthy that the body weight used in the guidelines is based on IBW to avoid administering large volumes to very obese patients [114,198-200]. Over-resuscitating septic patients could increase mortality and induce volume overload complications. In a recent retrospective study, using more than 50 mL/kg IBW was associated with higher mortality compared to the guideline-directed dosage [200].

In a retrospective study by Kuttab et al., failure to administer 30 mL/kg of IV fluids (based on actual body weight) during the first three hours was associated with a higher in-hospital mortality rate, regardless of the comorbidities, including obesity [201]. However, many studies show potential harm in administering 30 mL/kg based on actual weight, compared to other restrictive approaches [202]. The administration of a minimum of 30 mL/kg came from the average volume of fluid that was used in the ARISE, PROCESS, and PROMISE trials [114,160-162,203].

Due to the low-quality evidence, this recommendation of administering 30 mL/kg of fluids is downgraded from a strong recommendation with low-quality evidence in the 2016 guidelines to a weak recommendation with low-quality evidence in the 2021 guidelines [114,198]. Also, many recent studies challenge the current guidelines by showing that there is no difference in mortality between liberal and conservative approaches, and the liberal approach is not superior to the conservative approach [204-207]. Of interest, other studies show that liberal fluid resuscitation could be harmful and might increase mortality, LOS, or hypervolemia-associated complications, compared to a conservative approach. These studies also show that early initiation of vasopressors with conservative fluid resuscitation might be beneficial for reducing mortality and decreasing the incidence of hypervolemia complications [204,208-212].

Clinicians should be careful during fluid resuscitation and monitor the development of hypervolemia-related complications, such as pulmonary edema. More studies are encouraged to assess the effectiveness of early initiation of pressors with less fluid administration. Additionally, guiding initial fluid resuscitation by assessing fluid responsiveness and volume status should be investigated.

Having a sepsis protocol for clinicians who are not confident in managing high-acuity patients, such as septic patients, or who lack a deep understanding of physiology and pathophysiology, is crucial. However, for experienced clinicians who understand sepsis physiology and pathophysiology, it is responsible to encourage a personalized approach to each patient that may deviate from the standardized protocol based on the patient’s other co-morbidities. For example, the decision to use liberal or conservative initial fluid resuscitation should be based on many factors, such as the patient’s volume status, fluid responsiveness, heart pathologies, and presence of other comorbidities, rather than on a rigid rule of 30 mL/kg that fits every patient. This is supported by three trials developed after the EGDT trial by Rivers et al., including ARISE, PROCESS, and PROMISE. These three trials showed that, with more experienced clinicians who provide care influenced by EGDT, deviating from the protocol was not inferior to following it [114,160-162,203].

Fluid resuscitation in septic patients with hypervolemia due to CHF and ESRD: Fluid resuscitation is a cornerstone in the initial resuscitation of septic patients. However, there is a risk of developing hypervolemia-related complications, such as pulmonary edema, increased time of mechanical ventilation, the need for emergent dialysis, increased ICU LOS, and mortality in patients with ESRD, chronic kidney disease (CKD), or CHF. This concern stems from the fact that the 2021 sepsis guidelines do not include separate fluid resuscitation guidelines for this population. This is evident in reality when clinicians attempt to avoid administering the entire initial fluid bolus to septic patients with CHF or ESRD. As expected, there is low compliance with 30 mL/kg fluids during initial resuscitation, especially in patients with ESRD and CHF [114,201,213,214].

Many observational studies and clinical trials have shown that after administering the guideline-recommended fluid bolus (30 mL/kg), there is no significant difference between patients with ESRD or CHF and those without these comorbidities regarding the development of complications, such as ICU LOS, hospital LOS, mechanical ventilation time, and mortality, as discussed below [213-219]. This can be explained by the fact that in septic patients, even those with CHF or ESRD, some vasodilation occurs, creating a space for more fluids that are needed to support blood pressure. Those patients can present with clinical symptoms of hypervolemia, such as lower extremity edema, which reflects increased fluid volume in the interstitial space rather than the intravascular space.

Regarding patients with ESRD, in a case-control study, among patients who received 30 mL/kg or more of fluids, the differences in intubation rate, LOS in the ICU, hospital LOS, mortality, and need for urgent dialysis between ESRD patients and patients without ESRD were insignificant. Additionally, among ESRD patients, there was no significant difference between those who received 30 mL/kg or more and those who received less than that regarding mortality, LOS, and the need for urgent dialysis [213]. Other observational studies show similar results [215-217]. A secondary analysis of the PROCESS trial was conducted to investigate whether there is an increase in adverse outcomes in septic patients with ESRD when given the guideline-suggested fluid volume. It shows that administering 30 mL/kg or more to ESRD patients yields similar outcomes to those without ESRD [218].

In CHF, a case-control study showed that the use of 30 mL/kg or more of fluids during initial resuscitation does not increase the incidence of ICU admissions, mechanical ventilation rate, and ICU LOS compared to both patients without CHF who received 30 mL/kg or more and CHF patients who received less than 30 mL/kg [214]. Other observational studies show similar results [215,217,219-221]. Two systematic reviews and meta-analyses show that the administration of 30 mL/kg or more of IV fluids in septic patients who are at risk of hypervolemia, such as patients with ESRD and CKD, does not increase mortality, LOS, or mechanical ventilation incidence [222,223].

All of these previous studies suggest that guideline-directed fluid resuscitation in septic patients with ESRD or CHF is safe and does not cause increased mortality or a high rate of complications. However, these studies also show that liberal fluid resuscitation is not superior to conservative fluid resuscitation regarding mortality. Compliance with guideline-directed fluid resuscitation was not found to be associated with a decrease in in-hospital mortality compared to noncompliance in septic patients with high-risk hypervolemia. This confirms that liberal fluid resuscitation is not superior to the conservative approach [224]. This also questions the need to give guideline-directed amounts of fluid to septic patients with ESRD if it does not improve mortality.

Notably, some studies suggest that fluid resuscitation guided by guidelines may be detrimental. In contrast to previous studies, guideline-guided fluid resuscitation was found to be potentially harmful, as it was associated with a decrease in the rate of discharge home [225]. In a systematic review and meta-analysis by Khattar et al., guideline-directed fluid resuscitation in septic patients with ESRD was suggested to be potentially harmful [226].

A secondary analysis of the CLOVERS trial shows that in septic patients with advanced CKD, restrictive fluid resuscitation (median 424 mL) is associated with lower mortality by day 90, more days free from ventilator use at 28 days, and more days free from vasopressor use at 28 days, compared to liberal fluid resuscitation (median 2300 mL). In a subgroup of CKD patients who receive dialysis, the mortality rate is significantly lower in the restrictive group. In patients without CKD and those receiving CKD without dialysis, there was no significant difference regarding mortality in both the restrictive and liberal groups [227]. It is essential to notice that in the restrictive group, vasopressors were used more frequently, for a longer duration, and were initiated earlier [227]. This shows that conservative fluid resuscitation combined with early initiation of vasopressors is beneficial in septic patients with CKD requiring dialysis.

Given that the liberal fluid resuscitation approach in septic patients with a high risk of developing hypervolemia, such as patients with ESRD and CHF, is not superior to the conservative approach, and there is a potential harm of following the liberal approach, clinicians should be careful during the initial fluid resuscitation of septic patients with ESRD and CHF. Early initiation of vasopressors and giving small boluses, followed by reevaluation after each bolus, is encouraged. The management of septic patients with CHF and ESRD should be discussed in the future sepsis guidelines.

Fluid resuscitation beyond the initial fluid resuscitation in patients with sepsis and septic shock: Many septic patients may require more fluids after completing the initial fluid resuscitation due to persistent hypoperfusion. Fluid boluses beyond initial resuscitation could be beneficial by increasing hemodynamic parameters, such as MAP and CVP [52]. However, there is a concern that giving more fluid beyond the initial resuscitation fluid may cause harm and induce hypervolemia, which is found to be associated with higher mortality [228]. Fluid boluses beyond initial resuscitation might also negatively affect the PaO2/FIO2 ratio [52]. As a result, assessing fluid responsiveness is introduced to differentiate between patients who will benefit from fluid administration beyond initial resuscitation and those who will not.

According to the 2021 sepsis guidelines, dynamic fluid responsiveness tools should be used to guide fluid resuscitation beyond the initial resuscitation to avoid over- and under-resuscitation. Additionally, the guidelines recommend dynamic measures over physical exams and static tools, as a weak recommendation with very low-quality evidence [114]. These dynamic parameters include pulse pressure variation and stroke volume variation in response to a fluid bolus or a passive leg raise [114].

Fluid Resuscitation in Patients With Rhabdomyolysis

The destruction of the muscular tissue and the release of the intracellular enzymes and electrolytes into the bloodstream is known as rhabdomyolysis. This results in electrolyte disturbances, such as hyperkalemia, hyperphosphatemia, and hypocalcemia. It may also lead to increased serum levels of uric acid, creatine kinase (CK), and lactate dehydrogenase, as well as myoglobinemia and myoglobinuria. Subsequently, the patient may develop AKI and high anion gap metabolic acidosis [229-231].

The treatment of rhabdomyolysis consists mainly of removing the offending agent if it is present and fluid resuscitation to improve renal perfusion and enhance the excretion of the myoglobin accumulated in the renal tubules [229-231]. A recent systematic review and meta-analysis showed that fluid resuscitation in patients with rhabdomyolysis decreased the incidence of AKI and the need for dialysis, while bicarbonate and mannitol did not show a significant benefit [232]. In a study comparing LR and 0.9% NS in the treatment of doxylamine-induced rhabdomyolysis, no significant difference was found in potassium levels or the time to CK normalization [233,234]. Clinical trials and observational studies are encouraged to compare the various types of fluid in rhabdomyolysis resuscitation.

The Danish Society of Intensive Care Medicine (DSIT), the Danish Society of Anesthesia and Intensive Care Medicine (DASAIM), and the Scandinavian Society of Anesthesiology and Intensive Care Medicine (SSAI) Clinical Practice Committee (CPC) suggest early fluid resuscitation to prevent rhabdomyolysis-induced AKI. They also suggest using crystalloids rather than colloids. These recommendations are weak and based on low-quality evidence. In contrast, no recommendations were made to suggest one over the other for balanced crystalloids versus isotonic saline [235,236].

The American Association for the Surgery of Trauma (AAST) Critical Care Committee recommends the use of either saline (0.9% or 0.45%) or LR solution in rhabdomyolysis with a starting rate of 400 mL/hour and a goal of urine output of 1-3 mL/kg/h and up to 300 mL/h [237]. Notably, all previous guidelines advise against the routine use of sodium bicarbonate and diuretics in the management of rhabdomyolysis [229,232,235-237].

Fluid Resuscitation in Patients With DKA

Fluid resuscitation is the most crucial initial step in the management of patients with DKA due to the hyperglycemia-induced osmotic diuresis and subsequent dehydration. However, the fluid resuscitation rate and the type of fluid in the treatment of DKA remain heterogeneous worldwide [238,239]. Theoretically, fluid resuscitation with large volumes of NS in DKA patients may result in hyperchloremia and, subsequently, normal anion-gap metabolic acidosis, which may not affect the anion gap. However, it could delay pH normalization.

In an RCT, DKA patients who received NS had significantly higher serum chloride levels and significantly lower serum bicarbonate levels than those in the LR group. This clinical trial supports the theory that high volumes of NS in patients with DKA may cause hyperchloremic acidosis. However, the size of this clinical trial was small, with only 52 patients [240]. Of interest, in another RCT with similar patient volume, there was a nonstatistically significant delay in the time to pH normalization in DKA patients who received NS compared to the LR group [241]. This trial suggests that the relative delay in pH normalization using NS, compared to LR, is not significant [242]. These contradictory results may be due to the small sample size, heterogeneity in DKA severity, and variations in treatment protocols.

In an analysis study including DKA patients from the SALT-ED trial and the SMART trial, balanced crystalloid administration was found to be associated with faster resolution of DKA and shorter time to insulin infusion discontinuation [9,128,243]. In a recent RCT, patients who received Plasma-Lyte-148 had faster DKA resolution compared to the NS group [244]. Having meta-analyses of these clinical trials was essential to have more substantial evidence. Recently, three systematic review and meta-analysis studies found that balanced crystalloid administration is associated with faster resolution of DKA compared to NS [245-247]. Based on these recent meta-analyses, balanced crystalloids have been shown to be more beneficial than NS and may reduce hospital stay in DKA patients.

The 2022 guidelines by the Joint British Diabetes Societies for Inpatient Care recommend using NS as the initial fluid of choice and switching to a 10% dextrose infusion when the blood glucose level is 14 mmol/L (252 mg/dL) to prevent hypoglycemia [239]. The American Diabetes Association (ADA) guidelines in 2024 recommend using NS or balanced crystalloids at a rate of 500-1000 mL/h for the first two to four hours to restore the volume deficit. After that, fluid resuscitation should be guided by the volume status, vital signs, fluid balance, and sodium concentration. Also, 5-10% dextrose in addition to the 0.9% NS should be used when blood sugar is less than 250 mg/dL (13.9 mmol/L) [248].

Fluid resuscitation in DKA patients with ESRD and CHF: Serial assessments of mental, renal, and cardiac status should guide fluid resuscitation in patients with ESRD or CHF to avoid hypervolemia. According to the 2022 guidelines of the Joint British Diabetes Societies for Inpatient Care, fluid replacement may not be necessary in DKA patients with ESRD, as they are unable to develop osmotic diuresis and are therefore not significantly dehydrated. In contrast, for those who build hypovolemia, boluses of 250 mL of NS or D10% could be tried with serial clinical assessments [239]. The 2024 ADA guidelines recommend using 250 mL boluses of NS or balanced crystalloids, with frequent clinical assessments in patients with ESRD or heart failure [248].

Fluid Resuscitation in Patients With Acute Pancreatitis

Patients with acute pancreatitis develop hypovolemia due to two reasons. First, decreased fluid intake and vomiting in patients with acute pancreatitis induce hypovolemia. Second, due to increased inflammatory markers, patients develop capillary endothelial damage, leading to fluid shift from the intravascular space to the interstitial space and the peritoneal cavity. Theoretically, this hypovolemia could lead to pancreatic hypoperfusion and necrosis, so it is reasonable to give those patients enough fluids to treat their hypovolemia and prevent pancreatic hypoperfusion [249,250].

According to the 2013 American College of Gastroenterology guidelines, early aggressive hydration is recommended during the first 12-24 hours with a rate of 250-500 mL/h to decrease blood urea nitrogen (BUN) as a strong recommendation with a moderate quality of evidence. Also, they recommend that more rapid repletion may be needed in patients with hypovolemia [250]. These recommendations were based on clinical trials and expert opinions [250,251].

In contrast, the 2022 WATERFALL trial showed that early, aggressive resuscitation could be harmful and associated with fluid overload. It also showed that moderate hydration could be as effective as early, aggressive hydration, with less risk of hypovolemia [252]. Also, many other studies and meta-analyses suggested against the use of aggressive hydration [253,254]. As a consequence, the 2024 American College of Gastroenterology guidelines suggest moderately aggressive resuscitation with a rate of 1.5 mL/kg/h (based on body weight) in patients without hypovolemia. Additional boluses (10 mL/kg) should be given for hypovolemia as a conditional recommendation with low-quality evidence [255]. Based on that, most patients will receive 3-4 L within the first 24 hours. Fluid resuscitation should also be early, within the first 24 hours, and the patient’s volume status should be reassessed frequently, aiming to lower the BUN level [255].

Regarding fluid type, many studies show that LR is superior to NS in the management of patients with acute pancreatitis because of the risk of NS-induced hyperchloremic metabolic acidosis and the benefit of LR-associated decrease in systemic inflammation. The 2024 American College of Gastroenterology guidelines suggest LR over NS in patients with acute pancreatitis as a conditional recommendation with a low quality of evidence [255-258].

Fluid Resuscitation in Patients With Acute Brain Injury/TBI

In this section, we discuss two separate areas of fluid administration in patients with acute brain injury: first, the use of fluids in resuscitation to achieve euvolemia or hemodynamic stability in critically ill patients; second, the use of fluid administration in the treatment of cerebral edema or increased intracerebral pressure (ICP) in those patients who develop increased ICP or cerebral edema after an acute cerebral injury.

Regarding fluid resuscitation in critically ill patients with acute brain injury, keeping an euvolemic status is associated with better outcomes [259-262]. Additionally, albumin and LR should be avoided in patients with acute brain injury. In aneurysmal subarachnoid hemorrhage (SAH), colloids were found to be associated with worse neurologic outcomes [263,264]. Interestingly, receiving synthetic colloids was found to be associated with decreased cerebral autoregulation [264].

In a secondary analysis of the SAFE trial, albumin 4% was associated with higher mortality in critically ill patients with TBI, compared to saline [265]. This increased mortality of albumin could be due to the proportional association between albumin administration and increased ICP in patients with TBI [266]. The increase in ICPs after albumin administration is unclear, whether it is due to the albumin molecule itself or to the hypotonicity of the 4% albumin (278 mOsm/kg) used in the SAFE trial. In an animal randomized controlled experimental study, hypotonic albumin 4% (278 mOsm/kg) administration was associated with increased ICP and CVP, compared to the isotonic albumin solution [267]. This means that the increased mortality in the SAFE trial is likely due to the hypotonicity of the 4% albumin used.

In contrast to albumin 4%, hypertonic albumin 20-25% was found to be beneficial in some observational studies and the studies that used the Lund protocol in the management of TBI [268-273]. However, albumin 25% did not have clinical benefits during fluid resuscitation in other types of brain injury, such as acute ischemic stroke (AIS), and was associated with unfavorable outcomes, such as pulmonary edema and symptomatic intracranial hemorrhage (ICH) [120]. No RCTs are discussing the use of hypertonic albumin 20-25% as a potential treatment in patients with TBI to decrease cerebral edema and mortality. RCTs are encouraged to investigate the potential benefit of hyperoncotic albumin in patients with brain injury. Based on the current data, albumin should be avoided in TBI. According to the ESICM, SFAR, and SFMU guidelines, crystalloids are preferred over albumin in critically ill patients with TBI due to the potential for increased mortality associated with albumin [22,45].

Similar to albumin 4%, LR (273 mOsm/L) is a hypotonic solution that may theoretically worsen cerebral edema [144-147]. Pre-hospital administration of LR was found to be associated with higher mortality compared to NS in patients with TBI [274]. In a secondary analysis of the BaSICS trial, Plasma-Lyte was found to be associated with higher mortality, compared to NS [275]. Despite its normal osmolality (294 mOsm/L), Plasma-Lyte is associated with worse outcomes. This could be because Plasma-Lyte’s buffer agents contribute an osmolality of 50 mOsm/L. and are prone to being consumed and converted to bicarbonate, which will be metabolized rapidly, especially in acidotic, critically ill patients. In a secondary analysis of the SMART RCT, the balanced crystalloid group that received LR or Plasma-Lyte had higher mortality or disposition to another facility in critically ill patients with TBI than the NS group [276]. The ESICM, SFAR, and SFMU guidelines recommend NS over balanced crystalloids in critically ill patients with TBI due to the risk of LR-induced hypoosmolality [22,45].

In a small RCT, there was no significant difference between Isofundine and NS in intracranial pressure, the development of intracranial hypertension, or mortality. The Isofundine group had a lower incidence of hyperchloremic acidosis compared to the NS group [277]. A smaller trial showed similar results [278]. These RCTs were very small. Also, both groups received HES, with the balanced fluid group receiving higher volumes of HES, which might have affected outcomes.

Regarding the use of fluids in decreasing ICP or cerebral edema in patients with increased ICP, hyperosmolar fluids play an important role. Given the rigid nature and the fixed volume of the skull, there are only three intracerebral components that fit in this fixed volume, including CSF, blood, and brain parenchyma. Any bleeding or cerebral swelling may lead to increased ICP, which can subsequently compress and ischemically damage brain cells [279]. Nonpharmacological therapies, such as CSF diversion, hyperventilation, craniotomy, external ventricular drain, decompressive craniectomy, and head-of-bed elevation, are used to reduce cerebral edema and ICP by altering intracranial pressure through mechanical and structural changes and do not affect the parenchymal swelling itself. In contrast, pharmacological therapies, such as hyperosmolar therapies, have a direct impact on cerebral edema [279,280].

Hyperosmolar therapies include mannitol and hypertonic saline at 3% and 23.4%. These hyperosmolar agents work by creating an osmolar gradient between the blood and the brain parenchyma. This osmolar gradient stimulates fluid shifting from the cerebral parenchyma to the bloodstream. Additionally, hyperosmolar solutions act as potent plasma expanders by drawing fluid from interstitial spaces into the bloodstream, thereby decreasing hemoglobin levels and blood viscosity, which may lead to reduced cerebral blood volume [281-283]. Some studies show that mannitol and hypertonic saline can be similarly effective in reducing ICP and cerebral edema. As a result, the use of mannitol or hypertonic saline usually varies between different trauma centers in the management of increased ICP in patients with TBI, which could be considered an acceptable variation [284-286].

In patients with elevated ICP or cerebral edema due to severe SAH, ICH, HE, TBI, or AIS, hypertonic saline administration was found to be associated with improved outcomes and brain oxygenation. There is also a potential effect on the ICP and cerebral edema following the administration of hyperosmolar therapy [283,287-298].

The Neurocritical Care Society guidelines suggest using hypertonic saline rather than mannitol for initial ICP and cerebral edema management in patients with TBI and ICH. They also recommended using either hypertonic saline or mannitol in patients with AIS or HE for the management of elevated ICP and cerebral edema. Additionally, symptoms-based dosing is recommended over sodium level-based dosing in patients with SAH for the management of cerebral edema and elevated ICP. All of these recommendations are conditional recommendations with low- or very low-quality evidence [280].

Due to the risk of developing hyperchloremia-induced AKI, hypertonic saline should be used cautiously [220,285]. The Neurocritical Care Society guidelines also suggest avoiding severe hypernatremia (upper limit 155-160 mEq/L) and severe hyperchloremia (upper limit 110-115 mEq/L) to decrease the risk of AKI, as a conditional recommendation with low-quality evidence [280]. However, many studies show that hypertonic saline-induced hyperchloremia might not be associated with the development of AKI [220,299]. Despite the questionable effect of hypertonic saline-induced hyperchloremia on kidney function, severe hyperchloremia should be avoided due to its adverse impact on acid-base balance and the potential development of metabolic acidosis [138].

Fluid Resuscitation in HRS

AKI may happen in cirrhotic patients with ascites for various reasons, including pre-renal azotemia, different types of shock, medication-induced AKI, acute tubular necrosis (ATN), and HRS. Additionally, AKI can be multifactorial in cirrhotic patients, and HRS-AKI may coexist with ATN or other causes of AKI, making differentiation difficult [300,301]. Historically, HRS has been classified into type 1 (acute) and type 2 (slowly progressive). However, the 2023 Acute Disease Quality Initiative (ADQI) and International Club of Ascites (ICA) guidelines recommend changing that historical classification of HRS into HRS-AKI, HRS-AKD, and HRS-CKD based on the duration and the time of onset of the kidney injury [300].

Cirrhotic patients with portal hypertension and ascites have increased production of vasodilators, such as NO, leading to splanchnic arterial vasodilatation and a decrease in the MAP. Subsequently, sympathetic innervation and activation of the renin-angiotensin-aldosterone system act as a reflex to increase MAP. This leads to sodium and water retention and vasoconstriction of many arteries, including the intrarenal arteries. These changes lead to a decrease in GFR and kidney function without structural or histologic changes [301,302].

Although the definitive treatment of HRS is liver transplantation, other treatment modalities should be initiated early to treat HRS-AKI. These treatment modalities may improve kidney function and decrease mortality. These treatment modalities include the combination of vasopressors with albumin and occasionally RRT. The timing of albumin and pressor initiation is the time of diagnosis. In contrast, the timing for RRT is controversial [63]. In general, RRT is indicated in HRS-AKI patients who do not respond to the pressor treatment, as well as in patients with dialysis indications similar to those in the general population, such as severe and/or refractory electrolyte disturbances, hypervolemia, acid-base imbalance, and symptomatic azotemia [62].

Another potential treatment for HRS-AKI is the transjugular intrahepatic portosystemic shunt (TIPS). It may increase CVP, preload, and cardiac output, thereby improving kidney function [303]. Some studies have shown improvements in kidney function after TIPS. However, these studies are small, and the evidence is limited [304,305]. A systematic review and meta-analysis showed that TIPS might have a potential mortality benefit in patients with HRS [306]. Clinical trials are encouraged to examine the effect of TIPS in the treatment of HRS [307].

The different definitions of HRS-AKI are based on the idea that HRS-AKI is a functional kidney injury caused by intrarenal vasoconstriction and is a diagnosis of exclusion. We can diagnose HRS-AKI by excluding other potential causes of AKI, especially pre-renal azotemia and ATN, through fluid challenge, renal ultrasound, urine analysis, and history of drug abuse or toxin intake. According to the 2015 definition by the ICA, HRS-AKI diagnosis criteria include the presence of cirrhosis with ascites and AKI, failing albumin challenge, absence of shock, absence of recent exposure to nephrotoxic substances, and absence of macroscopic signs of structural renal damage, such as microhematuria (> 50 RBCs per high power field), proteinuria (>500 mg/day), and normal renal ultrasound [308]. Both the 2021 AASLD and the 2018 EASL adopted that definition [62,63].

To initiate treatment earlier and ensure that all HRS-AKI cases are diagnosed early, the new guidelines from the ICA and the ADQI adopted a more liberal definition of HRS. The ICA and the ADQI developed new criteria to differentiate HRS-induced AKI from other types of AKI. These criteria include (a) having cirrhosis with ascites; (b) the presence of AKI; (c) the absence of strong evidence of other alternative causes of AKI; and (d) persistent elevated creatinine or a decrease in urine output within 24 hours after fluid resuscitation, when clinically appropriate to give fluids [300]. The significant difference between the 2023 and 2015 definitions lies in the albumin challenge.

Albumin challenge to diagnose HRS: The old ICA recommendation requires failing the albumin challenge: withholding diuretics and administering 1 g/kg/day, up to 100 g/day, for 48 hours to improve kidney function. If kidney function does not improve after 48 hours of albumin administration, the HRS diagnosis is confirmed [308]. Both the 2021 AASLD and the 2018 EASL guidelines adopted the use of the albumin challenge to confirm the diagnosis of HRS [62,63]. These recommendations are based on expert opinion and the benefits of using albumin in other conditions, such as SBP and after LVP, as discussed earlier. In contrast, the 2023 ADQI and ICA recommendations oppose the albumin challenge as a requirement for diagnosing HRS-AKI [300]. The new recommendations only require the absence of response to fluid resuscitation within 24 hours.

This early HRS diagnosis after 24 hours, rather than 48 hours, allows earlier initiation of vasopressors, which might be beneficial. However, there is a concern that this may lead to overdiagnosis of HRS and initiation of pressor treatment in patients who do not need it [309]. In a prospective study supporting the use of the EASL guidelines, a significant number of patients showed improvement in their AKI with the albumin challenge at 48 hours, thereby avoiding exposure to terlipressin. Those patients could have been exposed to unnecessary pressor treatment if we followed the new ICA guidelines [310]. Clinical studies are encouraged to investigate the benefit of the albumin challenge in preventing unnecessary exposure to pressors and in delaying pressor use by 24 hours for patients who really need them, if we follow the old guidelines.

The big question will be as follows: does the benefit of early pressor initiation after 24 hours for those who need pressor treatment outweigh the risk of early initiation of pressors for those who have a chance of AKI improvement at 48 hours? In a secondary analysis of a clinical trial, serum creatinine was found to be a significant predictor of reversal of HRS-AKI [311]. This supports that early initiation of pressors is beneficial and may increase the chance of responding to the pressor before having severe kidney dysfunction and severely elevated creatinine [312]. In patients with ACLF, the eTerli RCT showed that early initiation of terlipressin after 12 hours of failing the albumin challenge led to earlier reversal of AKI and reduced short-term mortality [313]. These results support the ICA’s new guidelines. Additionally, the side effects associated with pressor initiation, such as pulmonary edema, can be managed if they develop in people who received terlipressin unnecessarily.

Fluid management in HRS: The ADQI and the ICA recommend initiating vasopressors with terlipressin as a first-line pressor, along with hyperoncotic albumin 20-25%, immediately after the diagnosis of HRS-AKI, with a strong recommendation grade A [300]. Both the 2021 AASLD and the 2018 EASL guidelines support the use of albumin in combination with a pressor for the management of HRS [62,63]. Albumin increases the CVP, preload, cardiac output, and renal perfusion. It also has anti-inflammatory and antioxidant effects. Moreover, it might also decrease NO levels, thereby resolving splanchnic arterial vasodilatation.

Avoiding hypervolemia is crucial in patients with HRS to prevent complications, such as pulmonary edema, pleural effusion, and recurrent ascites that may affect the hemodynamics and subsequently the kidney function. The ADQI and the ICA recommend closely monitoring volume status during HRS management and adjusting the albumin dosage daily accordingly. They also recommend immediate discontinuation of albumin if the patient is clinically hypervolemic as a best practice statement [300]. In contrast, the 2018 EASL guidelines recommended 20-40 g/day, while the 2021 AASLD guidelines recommend an albumin dose of 1 g/kg on day 1, followed by 40-50 g/day for the rest of the therapy duration [62,63]. Relying on the hemodynamics, volume status, and general clinical condition to adjust albumin dosage is appealing and recommended in the 2023 ADQI and ICA guidelines. Clinical studies are encouraged to define the appropriate dosage of albumin.

Pressor management in HRS: Pressors are used to reverse the splanchnic arterial vasodilation, thereby increasing MAP, decreasing renin-angiotensin-aldosterone activation, and increasing renal perfusion. Many clinical trials and systematic reviews show the benefit of pressors, such as terlipressin and norepinephrine, on mortality and kidney function in patients with HRS [314-319]. In patients with ACLF, terlipressin was superior to norepinephrine. Terlipressin was found to have better reversal of HRS, a higher survival rate, and a lower need for RRT [319]. 

Regarding pressor management in HRS, the ADQI and the ICA recommend using serum creatinine to adjust the telipressin dose and MAP to adjust the norepinephrine dose. Regarding terlipressin, increasing the dosage daily if the serum creatinine does not decrease by 25% from baseline is recommended as a strong recommendation, grade D. Regarding norepinephrine, increasing the dose of norepinephrine every four hours if MAP has not increased by 10 mmHg or more from the baseline is recommended as a strong recommendation, grade B [300].

Norepinephrine was once the alternative to terlipressin for HRS in the US. However, terlipressin has been FDA-approved for use in patients with HRS in the US since 2022, following the North American CONFIRM trial, which showed that terlipressin was more effective than placebo in improving kidney function in HRS type 1 patients [314].

According to the ADQI and the ICA, discontinuation of the vasopressor in HRS is recommended if the kidney injury is resolved; the kidney injury does not improve after 48 hours of the maximum dose of the vasopressor; RRT is initiated; there is a severe adverse reaction caused by the vasopressor; or the patient has been on the pressor for the maximum duration, which is 14 days. This recommendation was graded as a string recommendation, grade B [300].

## Conclusions

Fluid resuscitation plays a crucial role in maintaining homeostasis, enhancing tissue perfusion, correcting hemodynamic instabilities, and reducing inflammation in different clinical scenarios. On the other hand, excessive fluid resuscitation can lead to fluid overload, and certain types of fluids may have adverse effects on human physiology, potentially causing harm that should be avoided. Critical care and emergency medicine physicians should be aware of the physiological features of different types of fluids and how their varying chemical compositions may affect the human body’s homeostasis. It is also essential to be aware of the most recent guidelines for fluid resuscitation to avoid complications associated with fluid resuscitation. There are still many clinical gaps regarding fluid resuscitation in critically ill patients that require future clinical studies.
